# Efficacy of Self-Directed Learning in the Supracondylar Fracture Performance Improvement Module at an Academic Pediatric Orthopedic Institution

**DOI:** 10.1155/2018/7856260

**Published:** 2018-07-02

**Authors:** Eric W. Edmonds, John W. Kemppainen, Joanna H. Roocroft, John Munch, Tracey P. Bastrom

**Affiliations:** ^1^Rady Children's Hospital, San Diego, CA, USA; ^2^University of California, San Diego, CA, USA; ^3^Helen DeVos Children's Hospital, Grand Rapids, MI, USA

## Abstract

Maintenance of certification (MOC) within a medical society requires continuing medical education that demonstrates life-long learning, cognitive expertise, and practice-based self-assessment. This prospective study sought to evaluate whether a self-directed Practice Improvement Module (PIM) would improve pediatric orthopedic patient outcomes, thus demonstrating evidence of life-long learning (Part II MOC credit) in treating supracondylar humerus fractures. Six surgeons and 113 patients were included. There was no significant difference in actual fracture outcome before or after PIM at any level of surgeon experience regarding radiographic appearance or need for reoperation (*p* > 0.10). Junior staff demonstrated a statistically significant improvement in the percentage of time that marking the operative site was documented in the chart by the surgeon before (38%) and after (65%) PIM (*p* = 0.02). The self-directed education portion of the supracondylar fracture PIM led to modest improvement in documentation habits among junior staff, without impact on overall patient outcomes. Therefore, the PIM appears to be less useful in providing evidence for life-long learning as it relates to surgical outcomes (Part II MOC/CME), yet, it may directly benefit practice-based self-assessment (Part IV MOC), and the self-assessment and Personal Improvement Plan may be the most important portion of the PIM to improve outcomes.

## 1. Introduction

Maintenance of certification (MOC) within a medical society requires continuing medical education that demonstrates life-long learning, cognitive expertise, and practice-based self-assessment. These can be defined in four distinct parts [1]. Part I is “Evidence of Professional Standing” which can be defined as having state licensure to practice medicine. Part II is “Evidence of Life-Long Learning” which is defined as having Continuing Medical Education (CME) credits and Self-Assessment Examination (SAE). Part III is “Evidence of Cognitive Expertise” which is one's ability to secure and pass a recertification examination. Part IV is “Evidence of Practice-Based Self-Assessment” which is defined as clinical case review and performance improvement activity.

As part of the American Board of Orthopaedic Surgery (ABOS) MOC process, POSNA released a Practice Improvement Module (PIM) for supracondylar humerus fractures, which was initially intended to satisfy the MOC requirement for practice-based self-assessment (Part IV MOC), but at the time of this study it was approved by the ABOS for Part II MOC credit [2]. The POSNA PIM is comprised of three sections. Section 1 consists of 33 questions pertaining to the preoperative evaluation, treatment, and follow-up of operatively treated supracondylar humerus fractures. The process involves the surgeon collecting specified data on 10 consecutive cases of supracondylar fractures treated surgically via the ABOS Scribe database. The surgeon then receives a report from the ABOS, comparing their responses to others that have submitted the same data. From this information, the surgeon can then begin Section 2 of the PIM, which involves the development of a Personal Action Plan. This plan is centered on areas of improvement highlighted by comparing their entered data to other ABOS diplomats. They are then directed to complete a CME activity. There are currently two methods to obtain the CME required to move onto Section 3: Option #1, which requires the completion of 4 out of 6 items (see Supplementary Appendix A), or Option #2, attending a POSNA annual meeting breakout session. Once this has been completed, then the surgeon would implement their Personal Action Plan and collect data on 10 subsequent cases to assess how his or her practice has changed.

Since the PIM process is intended to provide MOC credit (whether it is Evidence of Life-Long Learning or Evidence of Practice-based Self-Assessment), it is reasonable to assess the process and to validate its purpose. The POSNA PIM is one of the first Practice Improvement Modules to be approved for utilization and future PIM development will likely be based on the success of this first attempt. This study assesses the ability of the self-directed standardized CME portion of the PIM system to affect clinical outcomes among junior and senior staff at a level-one pediatric trauma center, attempting to validate its ability to demonstrate either life-long learning or practice-based self-assessment. We asked the following question: Does the POSNA-developed supracondylar humerus fracture PIM self-directed standardized CME improve pediatric orthopedic patient outcomes with this condition, thus demonstrating evidence of life-long learning for the surgeon (evidence for Part II MOC credit)?

## 2. Materials and Methods

A prospective study of surgeons treating supracondylar fractures operatively at a single institution was carried out over an enrollment period of 14 months (October 2012 to December 2013). Fractures treated by both junior (less than 2 years in practice) and senior staff were enrolled, with each surgeon confirmed to have completed at least 10 supracondylar humerus fracture surgeries prior to the study, to negate discrepancies in experience level [3]. The methodology of the PIM was altered for the purposes of this study and the surgeons were blinded to the questions and measures being obtained. Therefore, they did not fully participate in the transition between Sections 1 and 2 of the PIM, meaning that no improvement opportunities were identified and therefore no Personal Action Plans were developed. Instead, in an attempt to keep the surgeons blind to the study outcomes, after they had performed the initial 10 surgeries included in the study, the surgeons then underwent a self-driven computer-based education by completing 4 of 6 CME activities established by the PIM (see Supplementary Appendix A), as the official instructions were that only 4 were required and the remaining two items are courses to attend and are difficult to coordinate for study purposes. As intended in the PIM, the CME activity was self-paced and standardized, and no posttest or verification was completed.

Section 3 of the PIM was then completed with data collection and enrollment continuing after the surgeon education sessions to reach a target of 10 post-PIM cases for each surgeon. Even though the surgeons were the subjects being studied, our Institutional Review Board asked that each patient being reviewed be consented prospectively for their data to be collected. Research staff members then audited the enrolled cases from Sections 1 and 3. The questionnaire included in the POSNA PIM (Supplementary Appendix B) was utilized as the data collection sheet. Additionally, since the study was conducted at an academic institution with multiple midlevel and resident providers, data on whether the treating surgeon dictated the note was also recorded. The two questions pertaining to alignment on X-ray were answered by one of the surgeon authors who was not participating in the enrollment of cases, so that none of the participating surgeons were unblinded to the PIM questionnaire or measures of outcome.

Descriptive statistics before to after PIM education data were calculated to determine completeness of the record according to the PIM. Clinical (ROM compared to contralateral, limb alignment compared to contralateral) and radiographic (anterior humeral line and AP alignment) outcomes that were part of the PIM questionnaire were also compared before and after PIM. Furthermore, documentation and outcomes were compared by level of experience (junior versus senior staff). Proportions were compared utilizing chi-square analysis and all analyses were performed utilizing SPSS v. 12 (SPSS Inc., Chicago, IL).

## 3. Results

Six surgeons (4 junior and 2 senior) treated adequate numbers of supracondylar fractures for data before and after PIM session and 113 patients were included. There were 22 patients (19%) treated by senior staff before PIM education and 22 patients (19%) treated by senior staff after PIM education. There were 34 patients (30%) treated by junior staff before PIM and 35 (31%) treated after PIM education. Thus, a total of 56 patients (50%) were treated prior to staff PIM education and 57 (50%) were treated after staff PIM education. There were no significant differences in the proportion of times where the treating surgeon was the originator of the clinic notes before and after PIM (*p* > 0.05).

Before PIM, 27/56 (48%) of patients had at least a 6-week minimum follow-up visit. This increased to 37/57 (65%) after PIM education, although this was not statistically significant (*p* = 0.073). Detailed information of chart audits based on the PIM questions is presented in Table 1.

There was no significant difference in actual fracture outcome before and after PIM at any level of surgeon experience regarding radiographic appearance or need for reoperation (*p* > 0.10). Outcomes based on level of training, regardless of PIM status, are demonstrated in Table 2. Posttreatment radiographs demonstrated that the anterior humeral line passed through the central 1/3 of the capitellum in 70% of the junior staff cases and in 53% of the senior staff cases (*p* = 0.064). Alignment on the AP radiograph was anatomic within 5 degrees in 80% of the junior staff cases and in 93% of the senior staff cases (*p* = 0.051). For patients with available data, range of motion was contralateral within 10 degrees in 85% of the junior staff cases and in 63% of the senior staff cases (*p* = 0.095). Of the patients with available data, only one had documented clinical alignment that was not contralateral within five degrees.

Junior staff demonstrated a statistically significant improvement in the percentage of documented that the operative site was signed: from pre- (38%) to post- (65%) PIM (*p* = 0.02). They also demonstrated a nonsignificant increase in the documentation of limb perfusion prior to discharge (35%, before, and 66%, after; *p* = 0.10).

## 4. Discussion

At a level 1 academic children's hospital, the POSNA supracondylar fracture PIM self-directed education led to modest improvement in documentation habits among junior staff but did not affect overall clinical or radiographic outcomes. There are no comparative studies within orthopedics to help guide the utility of this particular PIM, but there is some evidence outside our field regarding the utility of Part II and Part IV MOC as well as PIMs.

Reflection regarding the ability of the PIM to affect patient outcomes requires a more basic level of assessing the MOC process, for which there has also been work looking into the utility of both MOC Part II and MOC Part IV [4]. Using a national database of physicians treating type 2 diabetes, Galliher and colleagues were able to demonstrate that both Part II and Part IV MOC correlated with greater improvements in care for patients treated by physicians participating in MOC in comparison to those treated by physicians not participating in MOC, suggesting that the PIM could affect patient outcomes. Yet, the authors note that they cannot make a direct association with the improvement in care and the participation in the Part II and Part IV exercises.

Previous study on the utility of a PIM has been performed, but the methods and conclusions are vague [5]. The American Board for Internal Medicine developed an osteoporosis PIM and then assessed the changes being seen by the participating physicians. Their methodology did not blind the participants from the outcome measures, which allowed them to review the results from all three sections of their PIM. Their conclusions were that the participating physicians could readily identify improvement opportunities within their practice and establish Personal Improvement Plans. Therefore, the authors concluded that the PIM assists the participant in evaluating their competency in practice-based learning and improvement (success regarding whether or not the PIM could change the quality improvement process for physicians).

Our study does not demonstrate an improvement in patient outcome based on the CME undertaken during the POSNA PIM but shows an improvement in documentation among young surgeons. It could be construed that improved “sign-your-site” documentation could be related to actually signing the extremity having surgery, which would then decrease the risk of wrong site surgery. However, our particular institution does not allow the patient to go back to the operating theater without the site being marked by the surgeon, so it truly is just an improvement in documentation by the surgeon concerning actions that they took. Moreover, the improved documentation of limb perfusion after PIM seen in our study could also represent an actual improvement in assessment of the patients prior to discharge, an improvement of care. A direct correlation remains difficult to state.

Although we had generally good clinical results and did not show a significant improvement in clinical outcomes after the self-directed learning portion of the PIM, this does not mean that there was not room for development. As a group, the surgeons did not achieve 100% compliance in any of the documentation or clinical measures outlined in the PIM. The authors of the PIM created its measured outcomes based on the best available evidence to maximize patient safety and clinical outcomes and, consequently, the goal of any orthopedic surgeon treating supracondylar humerus fractures should be to achieve 100% compliance in all measures. Based on this study, it appears that the self-directed standardized CME portion of the PIM alone does not achieve this goal. Perhaps the inclusion of the self-assessment and performance improvement plan would have allowed the self-reflection needed to see significant improvements in outcomes after the learning modules. Or perhaps the ability of a surgeon to seek specific CME related to their shortcomings in knowledge would improve patient outcomes rather than prescribing a standardized CME for all participants in the PIM process. Hence, the true value of this study may be that it demonstrates that standardized CME alone does not lead to measurable improvement in patient care.

One of the limitations of this study is the setting in which it was performed. Since it is an academic institution with frequent discussion of cases (both operative and nonoperative), as well as evaluation of surgical technique in a training environment, it may explain the lack of relationship between the PIM and clinical/radiographic outcomes in this study. A similar study, utilizing the same methods at a private practice without weekly conferences to assess the surgeons' radiographic outcomes, could have a completely different outcome from pre-PIM to post-PIM Section 2 CME. Future study on the efficacy of the PIM at impacting clinical outcomes at a nonacademic center may be valuable to confirm or refute this potential limitation.

Another limitation is the blinding of the surgeons to the transition from Section 1 to Section 2 of the PIM, eliminating the direct comparative feedback that would normally be available for identifying improvement opportunities, or the presence of a comparison group of surgeons that completed the Personal Improvement Plan. In order to prevent “gaming” of the results by the participating surgeons, it was imperative to keep the surgeons from knowing the measureable outcomes. This means that any changes seen from pre-PIM to post-PIM Section 2 self-study were purely the result of the CME undertaken and therefore accurately reflect changes related to the PIM (rather than changes related to an attempt to get a better score or demonstrating success in reaching the self-proclaimed goals of a Personal Improvement Plan). Admittedly, structured self-reflection of one's practice may more effectively drive clinical improvements than CME activities alone, and the omission of the Personal Improvement Plan may have tempered any potential benefits regarding clinical outcome. Perhaps the “gaming” of the results that we sought to prevent by blinding the surgeons is, in fact, the truly valuable component of the PIM that could lead to measurable improvements in patient care. However, after this study, we believe that the standardized CME portion (set in a general fashion by the PIM process and not related to a surgeons' Personal Improvement Plan) likely can be considered as ineffective in improving patient outcomes. A better methodology may be to allow surgeons to tailor their CME to meet their specific needs, based on their self-reflection.

Therefore, the next study will need to include the Personal Improvement Plan in order to determine whether the standardized CME can be useful in that setting. If it is not, then future PIMs will need to be modified to allow personal CME choices (rather than a standardized CME) that are based on the Personal Improvement Plan.

Another limitation is that the treating surgeons, as noted above, did not always perform the documentation themselves. Therefore, changes to documentation may not reflect changes in the practice of the treating surgeon. However, as with all academic centers, the attending surgeon is the owner of the chart and patient care. Therefore, from a medical-legal standpoint, the onus of documentation belongs to the attending surgeon, and with each note being cosigned, we believe that changes to the documentation do in fact represent changes to the treating surgeons' practice.

Currently, the ABOS intend to offer the supracondylar PIM for Part II MOC credit (Evidence of Life-Long Learning). This study demonstrates that self-directed learning in the absence of self-reflection on one's practice does not significantly change clinical outcomes but may modestly change documentation patterns among young surgeons. However, the PIM system does appear to provide evidence for practice-based self-assessment, since it directly records surgeons' documentation habits in real time for outside MOC committee review. Improvements in practice habits may represent an improvement in patient care, which is the importance of Part IV MOC criteria. Therefore, reconsideration by the ABOS to utilize the POSNA supracondylar fracture PIM for Part IV MOC credit may be warranted. Future development of PIMs and MOC credits within medical societies should stress the importance of self-assessment of physician practice to affect patient outcomes, and the development of personalized CME may be warranted to utilize the PIMs for Part II MOC credit.

## Figures and Tables

**Table 1:** Detailed response rates for PIM questions before and after PIM education, based on level of experience as well as for the cohort as a whole. Significant differences are noted via bold values; differences at alpha < 0.10 are in italics.

	Junior	Senior	All
*Preoperative assessment*			
Limb perfusion documented			
Before PIM	94%	95%	95%
After PIM	97%	91%	95%
Neuro deficit/exam documented			
Before PIM	97%	100%	*98%*
After PIM	86%	95%	*90%*
AP/lateral X-rays obtained			
Before PIM	97%	100%	98%
After PIM	86%	95%	90%
*Treatment assessment*			
Time of injury available to determine length of time between injury and treatment			
Before PIM	9%	18%	13%
After PIM	6%	9%	7%
Informed consent obtained and documented in op. note			
Before PIM	100%	95%	98%
After PIM	100%	98%	98%
Site signed and documented in op. note			
Before PIM	**38%**	100%	63%
After PIM	**65%**	86%	74%
Surgical pause performed and documented in op. note			
Before PIM	97%	77%	89%
After PIM	97%	91%	95%
Preop. antibiotics given and charted in op. note			
Before PIM	76%	86%	80%
After PIM	86%	86%	86%
Limb perfusion documented after reduction and fixation			
Before PIM	24%	14%	20%
After PIM	37%	5%	25%
Lateral pins only?			
Before PIM	76%	73%	75%
After PIM	69%	72%	70%
Degree of flexion in cast documented			
Before PIM	18%	18%	18%
After PIM	29%	18%	26%
Neuro deficit/exam documented prior to discharge			
Before PIM	44%	64%	52%
After PIM	54%	64%	58%
Limb perfusion documented prior to discharge			
Before PIM	*35%*	68%	48%
After PIM	*66%*	55%	61%
*Aftercare and outcome at final follow-up*			
Pins removed at 3 weeks?			
Before PIM	*76%*	73%	75%
After PIM	*51%*	76%	61%
Cast d/c at 3 weeks?			
Before PIM	68%	64%	66%
After PIM	46%	62%	52%
Limb perfusion documented at last f/u?			
Before PIM	85%	59%	75%
After PIM	89%	77%	84%
Neuro deficit/exam documented at last f/u?			
Before PIM	85%	82%	84%
After PIM	91%	95%	93%
On A/P X-ray, was alignment within 5 degrees anatomic?			
Before PIM	76%	95%	84%
After PIM	83%	91%	86%
On lateral, does anterior humeral line pass through central 1/3 of capitellum?			
Before PIM	74%	55%	66%
After PIM	66%	50%	59%
Patient has minimum 6-week f/u?			
Before PIM	41%	59%	*48%*
After PIM	57%	77%	*65%*
Physical exam documented elbow alignment at final visit?			
Before PIM	57%	31%	44%
After PIM	55%	35%	46%
Range of motion documented at final visit?			
Before PIM	76%	82%	79%
After PIM	83%	91%	76%
Time of return to unrestricted activity known?			
Before PIM	100%	92%	96%
After PIM	80%	94%	87%
Reoperation?			
Before PIM	0%	0%	0%
After PIM	5%^*∗*^	0%	3%^*∗*^

^*∗*^1 patient had reoperation for septic elbow.

**Table 2:** Summary of clinical and radiographic outcomes based on experience.

	Junior	Senior	*p* value	Combined
Anatomic AP alignment within 5 degrees	55/69 (80%)	41/44 (93%)	0.051	96/113 (85%)
Anterior humeral line passing through central 1/3 of capitellum	48/69 (70%)	23/44 (53%)	0.064	71/113 (63%)
ROM within 10 degrees of other side^*∗*^	17/20 (85%)	15/24 (63%)	0.095	32/44 (73%)
Clinical alignment within 5 degrees of contralateral upper extremity^*∗*^	19/19 (100%)	9/10 (90%)	0.345	28/29 (97%)

^*∗*^ *n* is reduced for these variables, as those with <6-week follow-up or those where the outcome of interest was not recorded in visit note are excluded from the analysis.

## Data Availability

The data used to support this study are not publicly available per the protocol approved by the local IRB. The consents signed by the patients for this study did not include a plan to make the data publicly available.

## Supplementary Materials

Supplementary 1 Supplementary appendix A: option 1, complete 4 of 6 of the following activities.

Supplementary 2 Supplementary appendix B: suparacondylar humerus fracture PIM.
