# Supplementary material for: Efficacy of Self-Directed Learning in the Supracondylar Fracture Performance Improvement Module at an Academic Pediatric Orthopedic Institution
Source: Adv Orthop. 2018 Jul 2;2018:7856260. doi: 10.1155/2018/7856260 (PMC6051276; doi:10.1155/2018/7856260)
Supplement: Supplementary 1 — Supplementary appendix A: option 1, complete 4 of 6 of the following activities. [file 7856260.f1.docx]

**Appendix A**

**Option 1, Complete 4 of 6 of the following activities:**

A. Read and review: AAOS Supracondylar Clinical Practice Guidelines found at: <http://www.aaos.org/research/guidelines/guide.asp>

B. Read and Review: J. Andy Sullivan, “Supracondylar Fractures of the Humerus in Children,” Orthopaedic Knowledge Online, Published 1/22/2007 <http://orthoportal.aaos.org/oko/article.aspx>?article=OKO_PED015

#article

C. Read and Review: M S Kocher et al, “Lateral Entry Compared with

Medial and Lateral Entry Pin Fixation for Completely Displaced

Supracondylar Humeral Fractures in Children. A Randomized

Clinical Trial.” *Journal of Bone & Joint Surgery - American Volume.*

89(4):706-12, 2007 Apr.

D. Attend: “The Difficult Pediatric Supracondylar Humerus Fracture: Tips and Techniques to Avoid Complications,” AAOS Annual Meeting Instructional Course Lecture.

E. Read and review: J M Abzug, M J Herman, “Management of Supracondylar Humerus Fractures in Children: Current Concepts,” *Journal of the American Academy of Orthopedic Surgeons*, 20(2): 69-77, 2012 Feb.

F. Read and Review: S T Mahan, C D May, M S Kocher, “Operative Management of Displaced Flexion Supracondylar Humerus Fractures in Children,” *Journal of Pediatric Orthopedics*. 27(5); 551-6, 2007 July-Aug.
