# Supplementary material for: Efficacy of Self-Directed Learning in the Supracondylar Fracture Performance Improvement Module at an Academic Pediatric Orthopedic Institution
Source: Adv Orthop. 2018 Jul 2;2018:7856260. doi: 10.1155/2018/7856260 (PMC6051276; doi:10.1155/2018/7856260)
Supplement: Supplementary 2 — Supplementary appendix B: suparacondylar humerus fracture PIM. [file 7856260.f2.docx]

**Appendix B**

**SUPARACONDYLAR HUMERUS FRACTURE PIM**

**Collect data for 10 consecutive cases**

**I. Pre-operative Assessment**

1. Last 3 digits of patient’s medical record number

2. Patient age (years):

3. What was the state of limb perfusion at presentation?

a. Radial pulse present

b. Radial pulse absent but hand perfused

c. Radial pulse absent and hand not perfused

d. Perfusion not documented

4. Was a neurologic deficit present at presentation? (check all that apply)

a. No deficit

b. Anterior interosseous nerve (median nerve)

c. Median nerve sensation

d. Posterior interosseous nerve (radial nerve)

e. Radial nerve sensation

f. Ulnar nerve motor function

g. Ulnar nerve sensation

h. Neurologic exam not documented

5. AP and Lateral radiographs of the distal humerus were obtained?

a. Yes

b. No

6. Fracture pattern?

a. Extension Gartland Type I

b. Extension Gartland Type II

c. Extension Gartland Type III

d. Flexion Type

e. Other

**II. Treatment**

1. Length of time transpiring between injury and definitive treatment?

a. Less than 6 hours

b. 6 – 12 hours

c. 12 – 18 hours

d. 18 – 24 hours

e. Greater than 24 hours

2. Was informed consent obtained and documented in medical record?

a. Yes

b. No

3. Was the site signed and documented in chart?

a. Yes

b. No

4. Was a surgical pause performed and documented in chart?

a. Yes b. No

5. Were pre-operative antibiotics given and charted?

a. Yes b. No

6. Operative Treatment

a. Closed reduction and percutaneous pinning b. Open reduction and pinning

c. Open reduction and screw fixation

d. Open reduction with plate and screw fixation

7. If pinning was performed, what pin configuration was used?

a. One lateral and one medial

b. Two lateral and one medial

c. Three lateral and one medial

d. Two lateral no medial

e. Three lateral no medial

f. Other

8. Intra-operatively, what was the state of limb perfusion following reduction and fixation?

a. Radial pulse present

b. Radial pulse absent but hand perfused

c. Radial pulse absent and hand not perfused d. Perfusion not documented

9. Intra-operatively, after reduction and fixation, if the hand was not perfused, did you explore the antecubital fossa?

a. Yes b. No

10. The elbow was immobilized in approximately what degree of flexion?

a. 40 – 60 degrees b. 61 – 90 degrees c. 91 – 120 degrees

d. Greater than 120 degrees

11. Were there any Intra-operative or procedural complications?

a. No b. Yes

12. If yes, please describe?

a.

13. Was the neurologic exam performed and documented in medical record prior to discharge from hospital? (check all that were tested)

a. Anterior interosseous nerve (median nerve)

b. Median nerve sensation

c. Posterior interosseous nerve (radial nerve)

d. Radial nerve sensation

e. Ulnar nerve motor function f. Ulnar nerve sensation

g. Neurologic exam not documented

14. Was limb perfusion recorded and documented in medical record prior to discharge from the hospital?

a. Hand of injured extremity was warm with capillary refill equal to contralateral, uninjured extremity

b. Hand of injured extremity had decreased perfusion compared to contralateral, uninjured extremity (less warm and/or delayed capillary refill)

c. Perfusion was not recorded

**III. Aftercare and Outcome At Final Follow-up (6 week Follow-up Necessary)**

1. If Kirschner wire fixation was used, when were the pins/wires removed?

a. Less than 3 weeks following the injury b. 3 weeks

c. 4 weeks d. 5 weeks

e. Later than 5 weeks following the injury

2. Was a pin tract infection or hypergranulation at pin sites present?

a. Yes b. No

3. When was immobilization discontinued and elbow range of motion begun?

a. Less than 3 weeks following the injury b. 3 weeks

c. 4 weeks d. 5 weeks

e. Later than 5 weeks following the injury

4. Limb perfusion at final follow-up (check all that apply)

a. Radial pulse present b. Radial pulse absent

c. Hand of injured extremity was warm with capillary refill equal to contralateral, uninjured extremity

d. Hand of injured extremity had decreased perfusion compared to contralateral, uninjured extremity (less warm and/or delayed capillary refill)

e. Perfusion was not recorded

5. Was a neurologic deficit present at follow-up? (check all that apply)

a. No deficit

b. Anterior interosseous nerve (median nerve)

c. Median nerve sensation

d. Posterior interosseous nerve (radial nerve)

e. Radial nerve sensation

f. Ulnar nerve motor function

g. Ulnar nerve sensation

h. Neurologic exam not documented

6. Considering alignment on physical exam at final follow-up, compared to the opposite elbow, was the injured elbow alignment:

a. Within 5 degrees of the contralateral, normal elbow

b. Greater than 5 degrees varus compared to the contralateral elbow

c. Greater than 5 degrees valgus compared to the contralateral elbow.

d. Elbow alignment not recorded

7. Considering range of motion on physical exam at final follow-up, compared to the opposite elbow, was the injured elbow total arc of motion:

a. Within 10 degrees of the contralateral, normal elbow

b. Within 10 to 20 degrees of the contralateral, normal elbow.

c. Within 20 to 30 degrees of the contralateral, normal elbow.

d. Within 30 to 40 degrees of the contralateral, normal elbow.

e. Contracture greater than 40 degrees compared to the contralateral elbow. f. Elbow range of motion not recorded

8. Were AP and Lateral radiographs of the elbow obtained?

a. Yes

b. No (skip to question #11)

9. On the AP x-ray, was the alignment?

a. In greater than 5 degrees of varus.

b. In greater than 5 degrees of valgus

c. Within 5 degrees of anatomic alignment.

10. On the Lateral x-ray did the anterior humeral line?

a. Pass through the central 1/3 of the capitellum.

b. Pass anterior to the central 1/3 of the capitellum.

c. Pass posterior to the central 1/3 of the capitellum.

11. Was re-operation performed for any indication?

a. Yes

b. No

12. If re-operation was performed, what was the indication?

a.

13. How long following the injury was the patient allowed to return to unrestricted activity?

a. 1 month

b. 2 months

c. 3 months

d. 4 months

e. Greater than 4 months
